# Compact portable multiphoton microscopy reveals histopathological hallmarks of unprocessed lung tumor tissue in real time

**DOI:** 10.1002/tbio.202000009

**Published:** 2020-08-21

**Authors:** Laura M. G. van Huizen, Teodora Radonic, Frank van Mourik, Danielle Seinstra, Chris Dickhoff, Johannes M. A. Daniels, Idris Bahce, Jouke T. Annema, Marie Louise Groot

**Affiliations:** ^1^ Faculty of Science, Department of Physics, LaserLab Vrije Universiteit Amsterdam Amsterdam Netherlands; ^2^ Department of Pathology Amsterdam Universities Medical Center/VU University Medical Center Amsterdam Netherlands; ^3^ Femto Diagnostics B.V. Uithoorn Netherlands; ^4^ Department of Surgery Amsterdam Universities Medical Center Amsterdam Netherlands; ^5^ Department of Pulmonary Diseases Amsterdam Universities Medical Center Amsterdam Netherlands

**Keywords:** autofluorescence, instant pathology, label‐free, lung tumor, portable microscope, second harmonic generation, third harmonic generation

## Abstract

During lung cancer operations a rapid and reliable assessment of tumor tissue can reduce operation time and potentially improve patient outcomes. We show that third harmonic generation (THG), second harmonic generation (SHG) and two‐photon excited autofluorescence (2PEF) microscopy reveals relevant, histopathological information within seconds in fresh unprocessed human lung samples. We used a compact, portable microscope and recorded images within 1 to 3 seconds using a power of 5 mW. The generated THG/SHG/2PEF images of tumorous and nontumorous tissues are compared with the corresponding standard histology images, to identify alveolar structures and histopathological hallmarks. Cellular structures (tumor cells, macrophages and lymphocytes) (THG), collagen (SHG) and elastin (2PEF) are differentiated and allowed for rapid identification of carcinoid with solid growth pattern, minimally enlarged monomorphic cell nuclei with salt‐and‐pepper chromatin pattern, and adenocarcinoma with lipidic and micropapillary growth patterns. THG/SHG/2PEF imaging is thus a promising tool for clinical intraoperative assessment of lung tumor tissue.
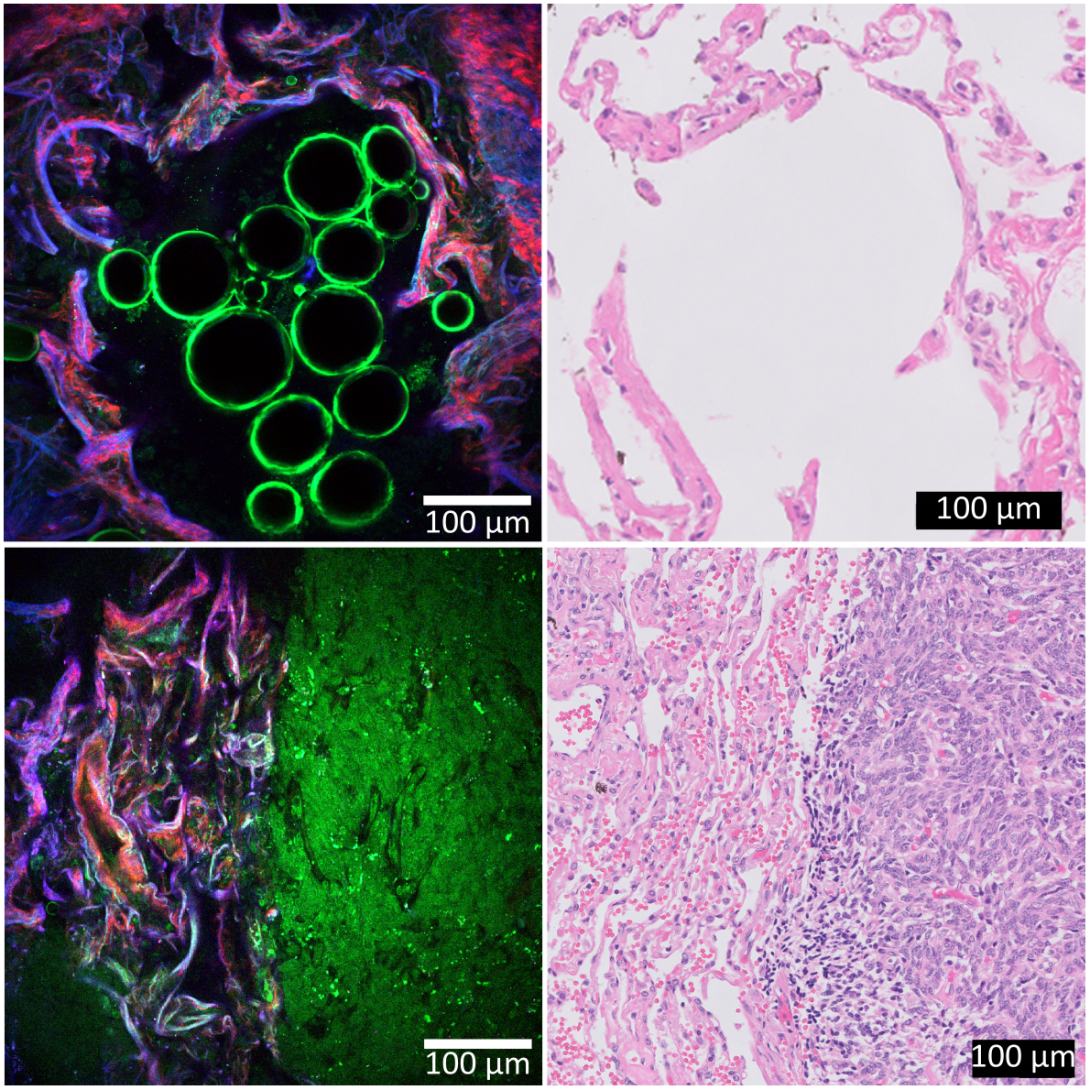

## Graphical abstract

A portable label‐free real‐time *ex‐vivo* microscope is used to investigate fresh unprocessed lung tumor tissue by generating third harmonic generation (THG), second harmonic generation (THG) and two‐photon excited fluorescence (2PEF) images, and comparing those images with standard HE histology images. Cellular structures (THG), and collagen (SHG) and elastin (2PEF) fibers can be distinguished, revealing important histopathological hallmarks (cell morphology and tissue architecture) for the assessment of lung tumor tissue.

**Abbreviations**
2PEF: two‐photon excited fluorescence
EVG: elastica‐Van Gieson
HE: hematoxylin and eosin
SHG: second harmonic generation
THG: third harmonic generation

## 1 INTRODUCTION

Lung cancer is the most common cause of cancer death, with 1.76 million deaths worldwide in 2018 [1]. Early stages of lung cancer (stage I and II) are usually treated with curative intent, which involves either surgical resection or radiotherapy, followed by adjuvant chemotherapy in selected cases. Even in stage III (large tumor or mediastinal lymph node involvement) surgery can play a role, usually after induction treatment with chemo‐ and/or radiotherapy. If surgery is part of the treatment plan, it is crucial to achieve a radical resection, with resection margins free of tumor. Rapid assessment of the resection margin or the type of the lesion might reduce operation time, and will potentially reduce complications and adverse effects.

Also, in the earlier stage accurate lung tumor tissue diagnosis is essential for optimal treatment allocation. Current lung tissue sampling techniques have their limitations often resulting in the acquisition of many biopsies that might lead to prolonged procedures, increasing the risk of complications or patient discomfort. Therefore, immediate feedback of lung tumor tissue sample adequacy is desired. Currently, the only available technique to provide fast feedback about the nature of the excised tissue and resection margins during surgery is fresh frozen section analysis, which provides histological information about frozen lung tissue slices, but which usually takes at least 20 minutes and requires a labor intensive workflow [2, 3]. An easy method to accelerate this process is thus required.

Multiphoton microscopy, including third and second harmonic generation (THG/SHG) and multiphoton excited autofluorescence (MPEF) generates images of unprocessed tissue with high, sub‐cellular resolution within seconds, and has been suggested to be a promising clinical tool for rapid histopathology [4]. This imaging technique is noninvasive, label‐free, and reveals 3D information due to intrinsic depth sectioning. The three imaging modalities provide complementary information: THG contrast originates from interfaces and inhomogeneities, revealing for example membranes of cells and their cell nuclei, and lipid bodies [5, 6, 7, 8, 9, 10, 11, 12]; SHG contrast comes from noncentrosymmetric molecules, such as collagen fibers [13, 14, 15, 16, 17, 18]; and MPEF contrast arises from autofluorescent molecules and structures, including cellular fluorophores such as flavin adenine dinucleotide (FAD), nicotinamide adenine dinucleotide (NADH) and extracellular fluorophores such as collagen and elastin fibers [19, 20, 21, 22, 23].

THG, SHG and MPEF microscopy has already been applied to lung tissue, in both animal and human tissues. Most studies have used SHG and/or two‐photon excited autofluorescence (2PEF) microscopy [24, 25, 26, 27, 28, 29, 30, 31, 32], and only a few studies used THG microscopy [33, 34, 35]. To the best of our knowledge, THG microscopy has not been applied to lung cancer tissue thus far, but only to normal and fibrotic diseased alveolar tissue [33, 34, 35]. These studies reported that THG is generated by micrometer‐size lipid bodies, cell nuclei, residual air, and elastin. The study by Sun et al found the combination of THG and 2PEF useful to reveal the alveolar structure, because of the correlating THG and 2PEF signals generated by elastic fibers [34].

The potential of the utilization of THG microscopy for cancer diagnosis has been shown for other tissue types [12, 36, 37, 38, 39, 40]. For example, Bopparts' research group demonstrated that THG signals reveal structural properties of extracellular vesicles, that is, microvesicles that are released by tumor cells and distributed throughout the microenvironment. Furthermore, they found that these vesicles are associated with breast cancer growth and metastases [41, 42, 43]. In addition, our own research group used the combination of THG and SHG microscopy to detect healthy and diseased states in fresh excised brain and breast tissue [4, 44, 45, 46]. Malignant and normal human brain tissue were successfully discriminated, based on histopathological hallmarks such as increased cellularity and nuclear pleomorphism. Furthermore, our group has quantitatively detected glioma infiltration by applying automated image analysis [45, 47, 48, 49].

In lung cancer research, most of the studies using SHG and 2PEF microscopy have focused on analyzing and quantifying the extracellular matrix. However, Jain et al used the combination of SHG and 2PEF imaging to visualize histopathological features to distinguish several subtypes of lung carcinoma [25]. They identified alveoli, bronchi, blood vessels, pleura, smokers' macrophages and lymphocytes, and showed a positive correlation of the amount of collagen and the degree of differentiation of adenocarcinoma. Jain et al concluded that SHG/2PEF microscopy has the ability to differentiate tumor from nontumorous lung tissue and to identify different tumor subtypes. They proposed that the simplest translation of this technique would be a benchtop microscope on a wheeled cart for assessment of ex‐vivo lung tissue.

In this study we tested for the first time such a transportable benchtop microscope: the FD1070nm microscope (Femto Diagnostics B.V.), which is a compact, portable, label‐free, real‐time imaging device that combines THG, SHG and 2PEF microscopy. We investigated *ex‐vivo* fresh unprocessed lung tissue (both tumor and nontumorous) and compared the THG/SHG/2PEF images with the standard histopathology images to identify the alveolar structures and histopathological hallmarks. We show that with the different imaging modalities we were able to differentiate between cellular structures (THG), collagen (SHG) and elastin (2PEF), and therefore were able to reveal important histopathological hallmarks (cell morphology, and general tissue architecture including collagen and elastin organization) for the assessment of lung tumor tissue.

## 2 MATERIALS AND METHODS

### 2.1 Ethics statement

All experimental procedures on human lung tissue were assessed by the medical ethical committee of the Amsterdam Universitair Medische Centra (Amsterdam UMC), approved by the Biobank Pathology Unit of the Amsterdam UMC, and in accordance with the Netherlands Code of Conduct for Research Integrity and the Declaration of Helsinki. All patients included in this study agreed, by signing an informed consent form, that their excised specimens could be used for scientific research.

### 2.2 Sample preparation

In this study, seven patients were included who underwent a lobectomy in the Amsterdam UMC. Five of these patients were diagnosed with adenocarcinoma, one patient with typical carcinoid, and one patient with a benign histology, namely an organizing pneumonia with abscess formation. After the operation, the resected lung tissues were transported to the pathology department of Amsterdam UMC. There, when possible, small pieces of tissue were excised, each with a maximum size of 1 × 1 × 1 cm^3^. This resulted in a total of 18 samples: six tumor samples from the core of the tumor and twelve nontumorous samples from an area distant from the tumor, whereof seven contained peripheral lung tissue and five bronchus wall tissue.

**FIGURE 1:**
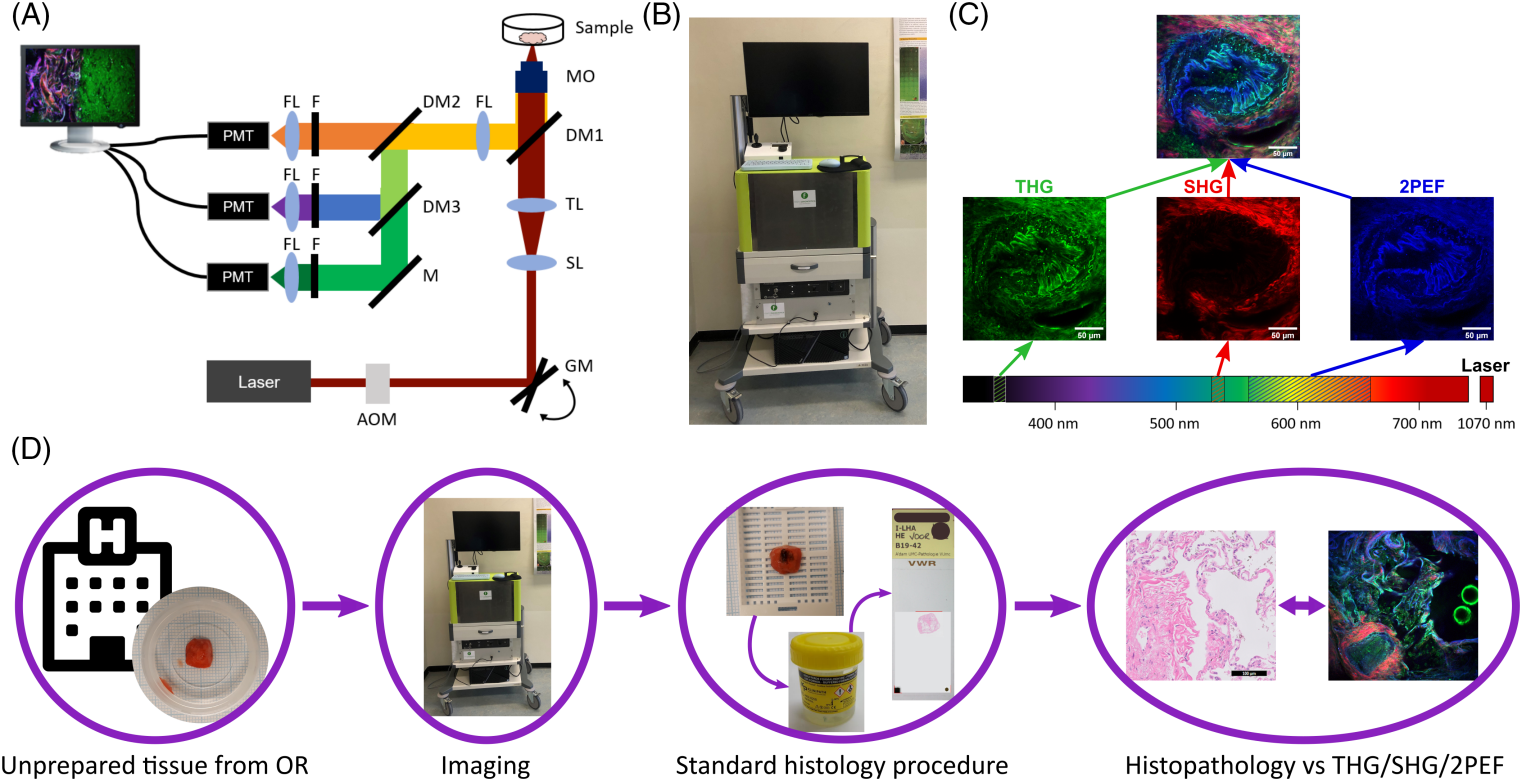
Measurement setup and procedure. A, Schematic overview of the setup, including acousto‐optic modulator (AOM), galvanometer mirrors (GM), scan‐ and tube lens (SL and TL), dichroic mirrors (DM), mirror (M), microscope objective (MO), focus lenses (FL), filters (F) and analogue photo‐multiplier tubes (PMT). B, Photo of portable FD1070 microscope. C, Third harmonic generation (THG), second harmonic generation (SHG) and two‐photon autofluorescence (2PEF) signals were separated by their detected wavelengths using appropriate filters, were depicted in green, red and blue, respectively, and were combined into one THG/SHG/2PEF image. D, Flowchart of the experiments: from lung tissue from hospital to THG/SHG/2PEF and histopathology images

The samples were placed in a sample holder (Ibidi, 35 mm diameter μ‐Dish with 0.17 mm thick glass bottom), and were immediately transported to the THG/SHG/2PEF microscope located in the Vrije Universiteit Amsterdam building next to the hospital (within 15 minutes walking distance). Each sample was imaged for 1 hour with the THG/SHG/2PEF microscope, subsequently fixated with 4% formaldehyde, and returned to the pathology department, where the samples were processed according to the standard histopathology procedure. This resulted in 3 μm thick hematoxylin and eosin (HE) stained slides that were scanned in order to easily compare the digital histopathology images with the acquired THG/SHG/2PEF images. (Figure 1D).

To obtain the best possible one‐to‐one comparison of the HE images and THG/SHG/2PEF images an ink dot was placed on the “nonimaging” side of the sample before it was fixated. Subsequently, in the pathology department, the first 100 μm of the “imaging” side of the sample was carefully sliced, and some slices were selected for HE staining. However, this was not always possible, and sometimes it was necessary to collect histology slices from deeper in the tissue, due to nonflat surfaces of the samples. In these cases, HE histology images that closely resemble the THG/SHG/2PEF images were used for comparison. Despite the differences in the levels examined, a histopathological pattern could be recognized in the imaged lung tissue which was characteristic for the case. Besides HE images, this manuscript shows two elastica‐Van Gieson (EVG) stained images, utilized to show relevant alveolar structures, but these were obtained from other nontumorous cases.

### 2.3 Image acquisition

To acquire THG/SHG/2PEF images a transportable FD1070 microscope (Femto Diagnostics B.V.) was utilized, that uses 1070 nm, 50 femtosecond pulses (Fidelity 2, Coherent) to generate nonlinear signals that are collected in backscatter direction by three detection arms (Figure 1A,B). Hence, both third harmonic generation (THG), second harmonic generation (SHG) and two‐photon excited autofluorescence (2PEF) signals could be detected at the same time by analogue photo‐multiplier tubes (H10720 and H7422, Hamamatsu) using appropriate filters: THG at 350 to 360 nm, SHG at 530 to 540 nm and 2PEF at 562 to 665 nm (Figure 1C). In the FD1070, a pulse picker is employed to select at 1 MHz bunches of 5 to 10 pulses (adjustable) out of the 70 MHz pulse train, matching the pixel dwell time. This way, the average power can be kept low, while retaining sufficient peak power to generate the nonlinear signals. Typically, a power of ~5 mW on the sample was used. A high numerical aperture (40×/1.3 NA, Nikon) oil‐immersion objective was used to generate a sub‐micrometer focus. The FD1070 microscope is an upward microscope, meaning that the sample needs to be placed above the objective. An 2D image of the sample was acquired by scanning the laser beam over the sample with galvanometer mirrors. 3D information of the sample was collected by scanning through the tissue in the vertical (*z*) direction by moving a motorized stage that contained the tissue holder, this also enabled larger range horizontal (*x* and *y*) navigation through the sample.

The THG/SHG/2PEF images were acquired with a LabView program (Femto Diagnostics B.V.). The LabView program provides several scanning modes, with different fields of view (FOVs), ranging from 50 to 500 μm, and it provides the possibility to automatically make a depth scan. For every FOV there is an “inspection” mode with an acquisition time of ~1 second, and a “high‐quality” mode with an acquisition time of ~3.5 seconds per image.

To enhance the weaker nonlinear signals, a gamma correction (with gamma factor 0.7) was applied to the images, and the contrast enhanced images were saved as RGB images (8‐bit BMP) with SHG displayed in red, THG in green and 2PEF in blue (Figure 1C). “Image J" software (ver. 1.52u, NIH, USA) was used to combine images of a depth scan in a depth stack (TIFF), and to create 3D projections and movies (AVI) of the depth scans.

## 3 RESULTS AND DISCUSSION

We imaged with THG/SHG/2PEF microscopy freshly excised, unprocessed lung tissue samples from seven patients undergoing lobectomy, and we compared the acquired THG/SHG/2PEF images with the histopathology images of these samples.

### 3.1 THG/SHG/2PEF images of nontumorous peripheral lung tissue compared with standard histopathology

**FIGURE 2:**
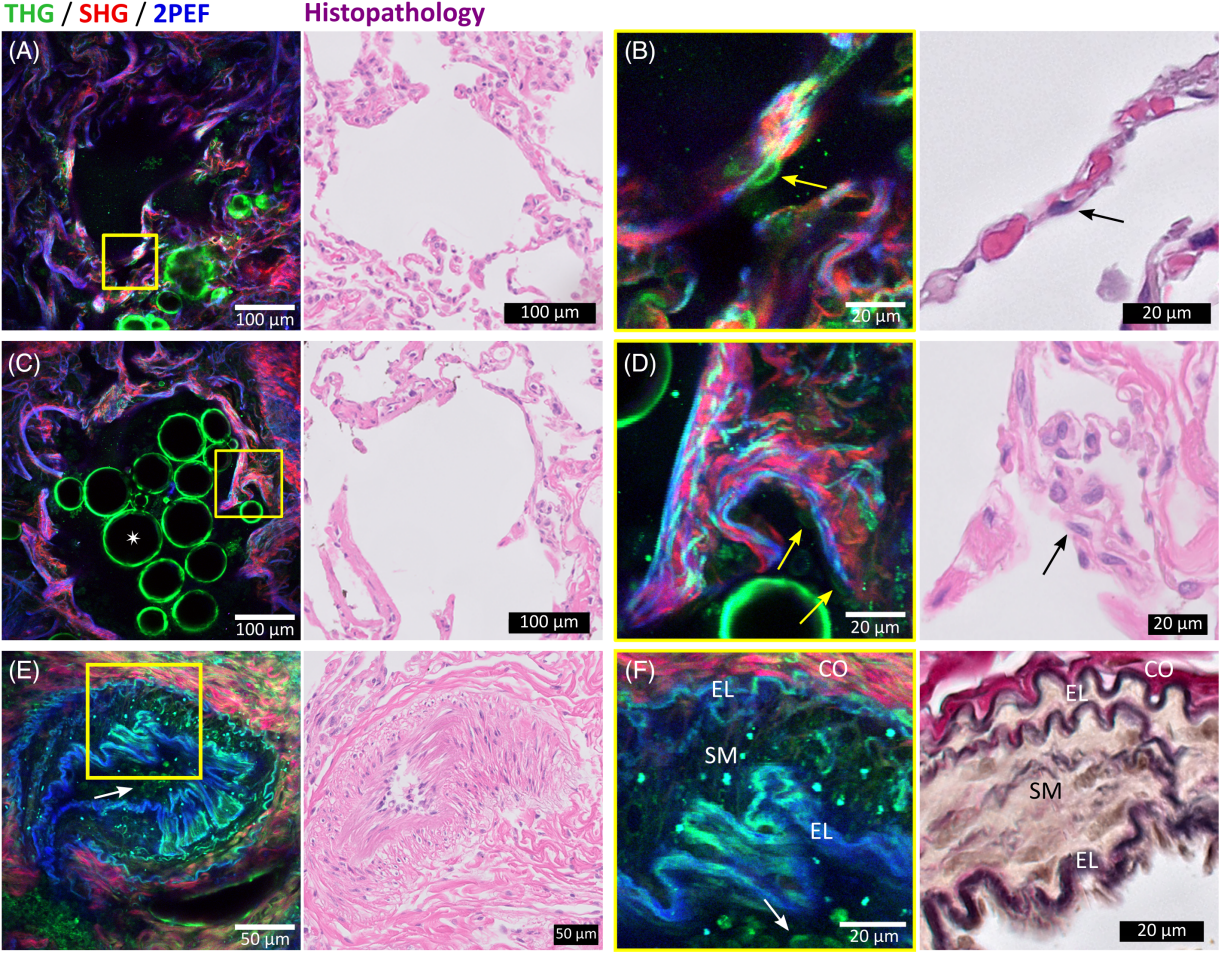
THG(green)/SHG(red)/2PEF(blue) images of nontumorous peripheral lung tissue (left) with corresponding histopathology images (right). (A‐D), Typical alveolar structures with alveolar wall including collagen (generating SHG), elastin (generating 2PEF), and pneumocytes (generating THG). Magnified images (of areas in yellow squares) show, (B), normal discontinuous thin alveolar wall and, (D), a thickened alveolar wall by fibrosis, with in both walls pneumocytes type II with flattened cell nuclei (yellow arrow in THG/SHG/2PEF image and black arrow in histology image). Contours of air bubbles inside alveolus generated THG signals (white star). (E,F), Different layers of an arterial wall formed by elastin (EL) visualized by 2PEF, smooth muscle cells (SM) visualized by THG, and collagen (CO) visualized by SHG. Erythrocytes inside the artery generated THG signals (white arrows). Acquisition time of THG/SHG/2PEF images is ~3.5 seconds per image using an average power of ~5 mW. Histopathology images include hematoxylin and eosin staining (A‐E), and elastica‐Van Gieson staining (F)

First, we imaged freshly excised, unfixed, structurally normal, lung tissue. Figure 2 shows THG/SHG/2PEF images (left) and corresponding standard histopathology images (right) of fresh nontumorous peripheral lung tissue, revealing typical peripheral lung structures including alveoli (Figure 2A‐D) and arteries (Figure 2E,F). Two important histopathological features of lung tissue are (a) the tissue architecture, made of collagen (type I and III are most abundant in the lung [50]) and elastin fibers which together form the alveolar skeleton of the lung and can be disturbed in the case of fibrosis and (b) cell morphology, whereas the cellular shape, size, and size of the nucleus are the most important features.

A normal alveolar wall (Figure 2A,B) is maximum two cell‐layers thick to facilitate close contact between erythrocytes (red dots in alveolar wall in HE histology image in Figure 2B) and air, and consists of alveolar cells: pneumocytes [51]. A magnification of the alveolar wall (Figure 2B) shows a pneumocyte type II (indicated with yellow arrow) of which the cell membrane generated THG signals, while the cell nucleus was silent in THG, and visible as a dark shadow within the cell. The cell nucleus is flattened, typical for normal alveolar epithelium.

**FIGURE 3:**
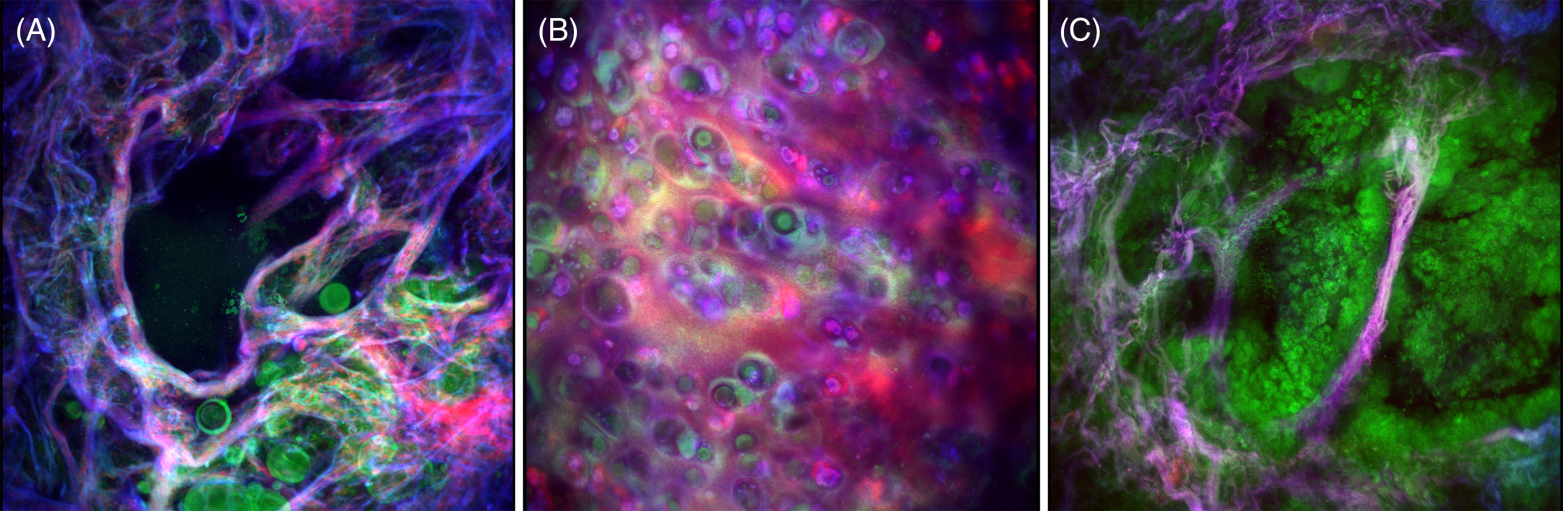
THG(green)/SHG(red)/2PEF(blue) top view images of 3D projections of (A), nontumorous peripheral lung tissue, (B), cartilage in nontumorous bronchus wall tissue, and (C), mucinous adenocarcinoma tissue. The 3D projections were generated from depths scans with a depth of 70 μm, (A,C), and 80 μm, (B). Corresponding videos of the 3D projections rotating 360° are shown in [Link], [Link], and S6

Besides pneumocytes, alveolar walls consist of collagen and elastin fibers [51]. The honeycomb architecture of collagen and elastin fibers is easy to recognize from the SHG and 2PEF images, respectively. Collagen and elastin fibers also generate THG signals, due to the water‐protein scaffold interfaces [34, 36]. However, the generated THG signals intensities are lower compared to the SHG and 2PEF signals, which causes the collagen fibers to be visible in red (mainly SHG) and elastin fibers in blue (mainly 2PEF). The elastin pattern in the alveolar wall appears discontinuous in 2D, because the lung tissue was collapsed *ex‐vivo*, which makes the elastin fibers wavy in 3D, which is also typically observed in histopathological 2D slides of lung tissue [52, 53]. In contrast, THG/SHG/2PEF microscopy provides 3D information, and a depth scan (Video S1) and 3D projection (Video S2 and Figure 3A) of this depth scan of these alveoli show that the elastic fibers are continuous in 3D.

The visualization of collagen is important to recognize fibrotic alveolar tissue, which is characterized by a thickened alveolar wall due to an excessive amount of collagen. Figure 2C shows an example of fibrotic alveolar tissue where parts of the wall are thicker compared to normal alveolar tissue. The magnified image (Figure 2D) shows the increased amount of collagen, clearly revealed by the SHG signal. Furthermore, in contrast to the normal structured alveoli, this fibrotic alveolar wall shows a 2D continuous elastin pattern, clearly revealed by the 2PEF signal. In the fibrotic alveolar wall, pneumocytes are also still visible, generating THG signals (indicated with yellow arrows). Furthermore, strong THG signals are generated by interfaces of round structures inside the alveolus (indicated with a white star), which are most likely air bubbles in protein‐ or lipid‐rich moisture such as surfactant. Using THG microscopy, air bubbles were also observed before by Pena et al and Débarre et al [33, 35]. Air bubbles are not present in the standard histopathology images due to processing of the tissue [33].

The corresponding HE stained histology images show the same pathological features in the normal and fibrotic alveolar tissue by revealing pneumocytes (indicated with black arrows) and collagen fibers, but in the HE histology images, elastin cannot be distinguished from collagen fibers. In contrast, elastin and collagen fibers are easy to distinguish using SHG and 2PEF microscopy, which enables recognition of the 2D discontinuous (Figure 2A,B) or 2D continuous elastin pattern (Figure 2C,D). To distinguish elastin from collagen in the histopathology, an additional staining, such as the EVG staining, is necessary.

In histopathology, an EVG staining is frequently used to distinguish different layers of arteries: elastin (in black), smooth muscle cells (in yellow), and collagen (in red) (Figure 2F right). Without the use of any staining, those three layers can be distinguished in the THG/SHG/2PEF images (Figure 2E,F left) because elastin, smooth muscle cells, and collagen give rise to 2PEF, THG and SHG signals, respectively. This clear separation of elastin, smooth muscle cells, and collagen makes it easy to recognize arteries in the THG/SHG/2PEF images. Furthermore, THG/SHG/2PEF images reveal biconcave disc‐shaped structures of 7 to 8 μm in diameter (Figure 2E,F indicated with white arrows) inside the artery, which meet the descriptions of erythrocytes [51], and it has been shown before that erythrocytes generate THG signals [54].

### 3.2 THG/SHG/2PEF images of nontumorous bronchus wall tissue compared with standard histopathology

**FIGURE 4:**
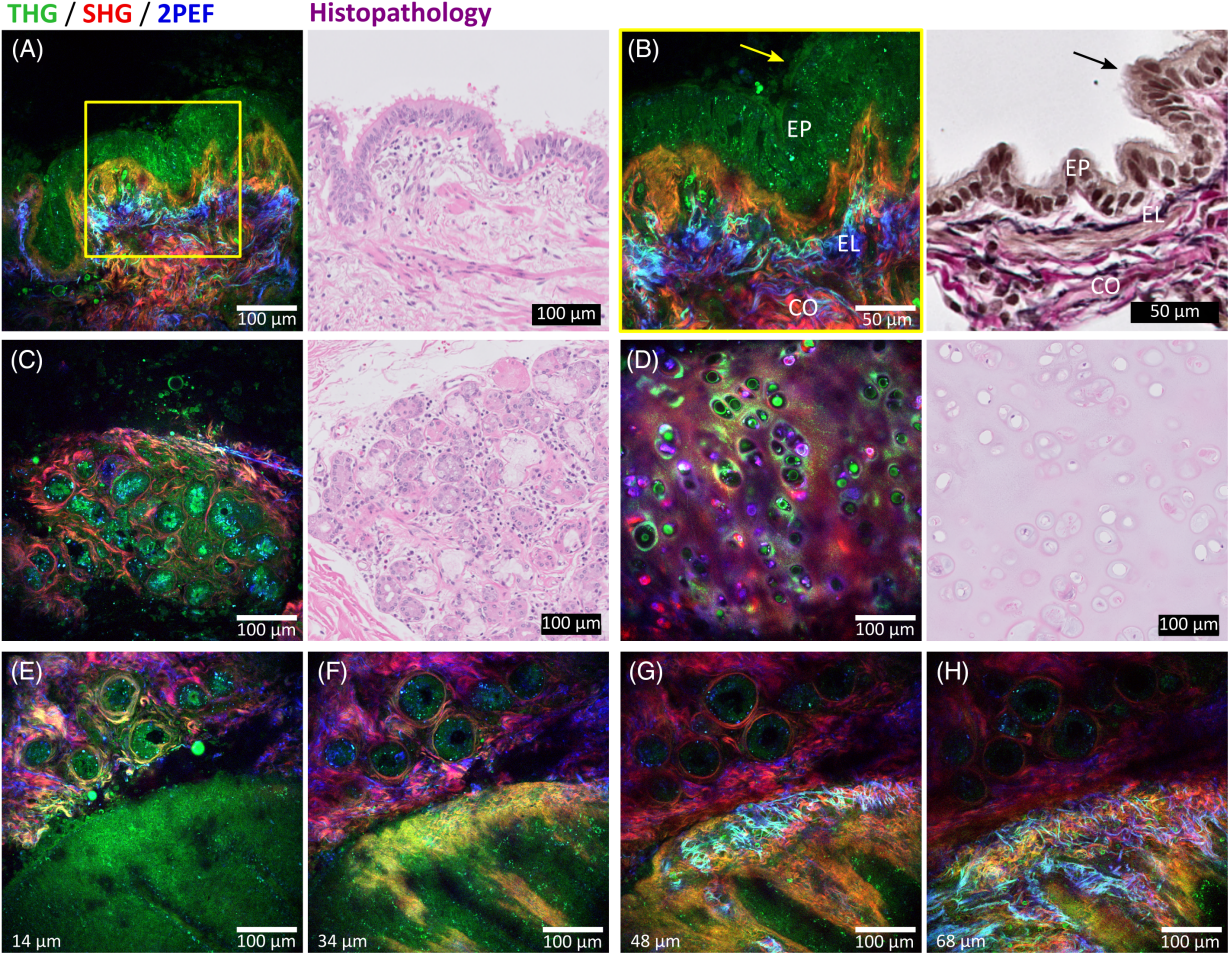
THG(green)/SHG(red)/2PEF(blue) images of nontumorous bronchus wall tissue with corresponding histopathology images. A,B, Different layers of the bronchus wall: epithelial cells (EP) generating THG, collagen (CO) generating SHG and elastin (EL) generating 2PEF. B, Magnified image (of area in yellow square) showing the brush border with cilia (yellow arrow in THG/SHG/2PEF image and black arrow in histology image). C, Glandular tissue. D, Cartilage tissue. E‐H, Images from a depth scan of up to 100 μm show the bronchus wall layers and glands at different depths. Acquisition time of THG/SHG/2PEF images is ~3.5 seconds per image using an average power of ~5 mW. Histopathology images include, A,C,D, hematoxylin and eosin staining and B, elastica‐Van Gieson staining

The bronchus wall consists of different layers: pseudostratified respiratory epithelium with ciliated brush border, lamina propria (including reticular [collagen III] fibers and elastin fibers) and submucosa (including seromucous glands and cartilage) [51]. The THG/SHG/2PEF images reveal each of the bronchus wall components, as shown in Figure 4. The first two layers are easy to identify (Figure 4A,B): the epithelial cells generated THG signals, while the lamina propria, containing collagen and elastin fibers, generated SHG and 2PEF signals, respectively. In accordance with the observations made in Figure 2 for the arteries, collagen and elastin are easy to distinguish in the THG/SHG/2PEF image (Figure 4A left), while in the histopathology, this distinction requires an EVG staining (Figure 4B right).

An important pathological hallmark of respiratory epithelium is the presence of the brush border, characterized by cilia, hair‐like structures that move debris and microbes up and out of the airways [55]. The magnified image shows that the brush border with cilia (Figure 4B, indicated by yellow arrow) is identifiable with THG microscopy in a way similar as in the EVG stained histology image (indicated by black arrow).

Both glands (Figure 4C) and cartilage (Figure 4D) are easily identifiable structures of the bronchus in the THG/SHG/2PEF images. Glands consist of groups of epithelial cells that generated THG signals, and are separated by collagen fibers that generated SHG signals. Part of the epithelium cells at the lumen side generated a more intense THG signal. Cartilage is easily identifiable by their large, rounded cells, that is, chondrocytes, that are lying in groups of up to eight cells [51]. The membranes of both the cells and their cell nuclei generated THG signals. Chondrocytes produce and maintain the extracellular matrix that consists of a mixture of proteoglycans and collagen type II [50, 51], which generated more homogeneous SHG signals instead of the fibrillar structures observed in the alveolar and bronchus wall tissue. The 3D composition of the chondrocytes in the extracellular matrix is displayed in a 3D projection of a depth scan shown in Video S3 and a top view of this 3D projection is displayed in Figure 3B.

Dependent on the orientation of the bronchus wall tissue, the bronchus wall layers were not always visible next to each other (as in Figure 4A,B), but also underneath each other or under an angle, which required a depth scan to identify all the layers. Video S4 shows a scan to a depth of 100 μm through the respiratory epithelium and lamina propria layers. Selected images at different depths are shown in Figure 4E‐H.

### 3.3 THG/SHG/2PEF images of tumor lung tissue compared with standard histopathology

**FIGURE 5:**
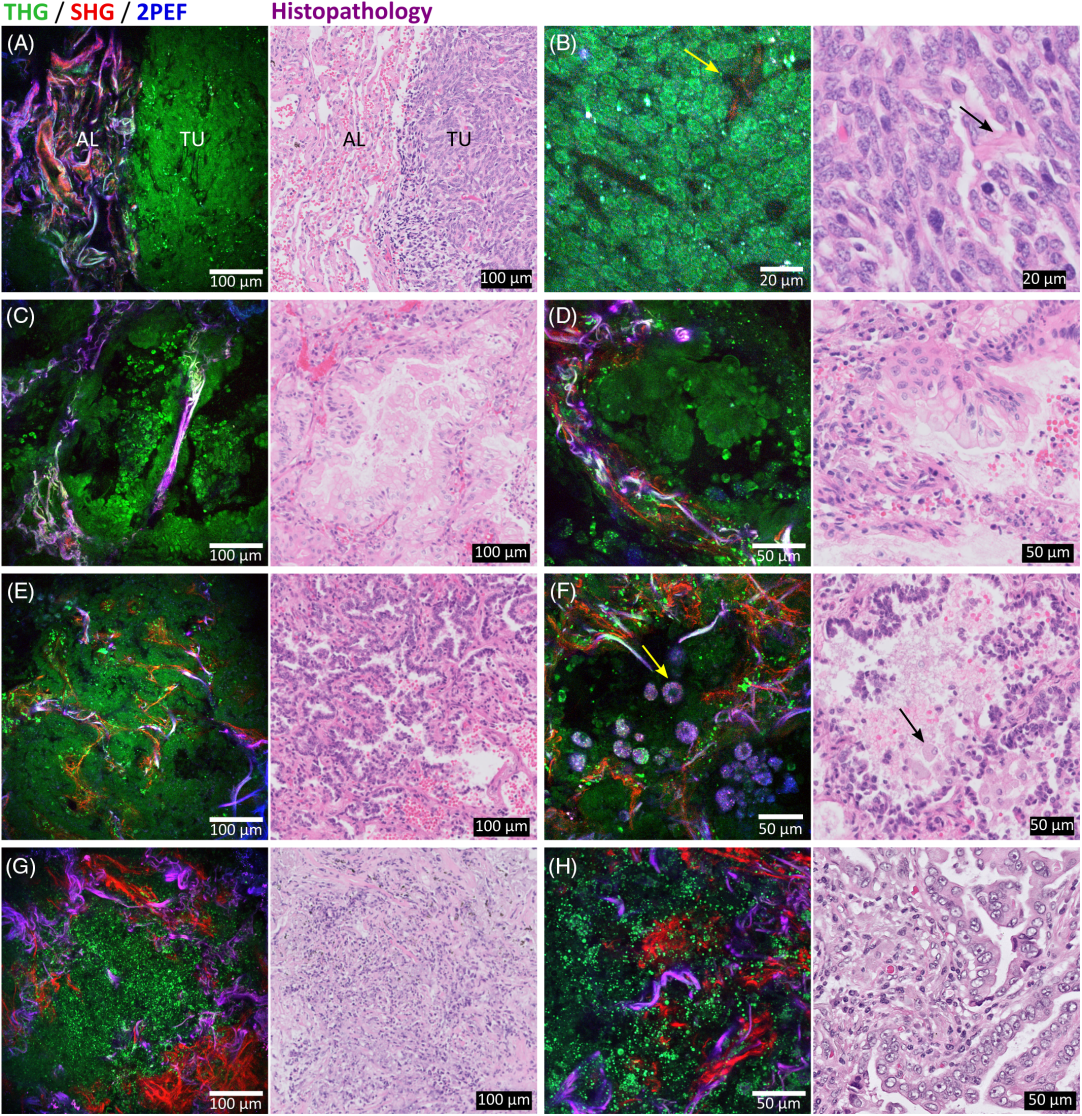
THG(green)/SHG(red)/2PEF(blue) images of various types of tumor tissues (left) with corresponding hematoxylin and eosin stained histopathology images (right). THG shows cellular structures, SHG collagen and 2PEF visualizes elastin and cytoplasm of pigmented macrophages. A,B, Typical carcinoid with solid growth pattern. A, Sharp transition area of preexistent collapsed alveolar tissue (AL) and tumor tissue (TU). B, Magnified image of tumor area shows minimally enlarged monomorphic cell nuclei with salt‐and‐pepper chromatin pattern, and shows a fibrovascular core (yellow arrow in THG/SHG/2PEF image and black arrow in histology image). C,D, Mucinous adenocarcinoma with lipidic and micropapillary growth pattern and partly intact alveolar wall. E,F, Adenocarcinoma with lepidic growth pattern. Alveoli were filled with cellular structures, including macrophages. Large cells with autofluorescent cytoplasm might be smokers' macrophages (yellow arrow in THG/SHG/2PEF image and black arrow in histology image, G,H, Mixed mucinous and nonmucinous adenocarcinoma with lepidic and acinar growth pattern with abundant number of inflammatory cells that contain bright THG spots. Acquisition time of THG/SHG/2PEF images is ~3.5 seconds per image using an average power of ~5 mW

Figure 5 shows a selection of THG/SHG/2PEF images of 4 of the 6 imaged tumor cases and the comparison with the standard HE histology images. The images show pathological hallmarks of different tumor types: typical carcinoid with solid growth pattern (Figure 5A,B), mucinous adenocarcinoma with lepidic and micropapillary growth pattern (Figure 5C,D), adenocarcinoma with lepidic growth pattern (Figure 5E,F) and mixed mucinous and nonmucinous adenocarcinoma with lepidic and acinar growth pattern (Figure 5G,H). In comparison to the nontumorous lung tissues (Figure 2), all tumor images show an abnormal amount of cellular structures, revealed by the THG signal generated by the cell nuclei, and show partly or completely destroyed alveolar walls.

Figure 5A shows a sharp transition area between preexistent alveolar tissue (left) and tumor tissue (right). The preexistent alveolar tissue was collapsed, recognizable by the denser honeycomb architecture of collagen and elastin compared to normal structured alveolar tissue. The tumor area is completely packed with cells with no preexistent alveolar structures, which is a pathological hallmark of a solid growth pattern of the tumor. Also note the collagen‐rich fibrovascular cores between the tumor cells, a histopathological hallmark of the carcinoid tumor. The magnified image (Figure 5B) shows a fibrovascular core generating SHG signals (indicated by yellow arrow), corresponding with the fibrovascular core in the HE histology image (indicated by black arrow). Furthermore, the magnified image shows that the tumor cells are so densely packed, that only the cell nuclear envelopes are visible with the THG modality. However, visualization of cell nuclear envelopes enables determination of important pathological features: the cell nuclear size and shape. The tumor cell nuclei in Figure 5B are minimally enlarged and monomorphic, meaning that they show little or no variation in morphology. Furthermore, the tumor cell nuclei have a salt‐and‐pepper pattern, appearing as dark purple spots in the HE histology image (Figure 5B right) and as bright spots in the THG channel (Figure 5B left). These bright THG spots in cell nuclei are also observed by Lin et al, who proposed that the high intensity signals could be generated by chromocenters, that is, condensed blocks of chromatin in cell nuclei, which is in agreement with the diagnosis of carcinoid [56].

In contrast to the solid carcinoid, the other tumor cases in Figure 5 show partly intact alveolar structures, as sporadically collagen and elastin fibers are present. Figure 5C,D show that the tumor cells are lining the preexistent alveolar walls (lepidic growth pattern) but also grow inwards the alveoli as “lollypops” (micropapillary growth pattern). This was a mucinous tumor, meaning that the cells are distinctive, tall and columnar, with cytoplasmic and extracellular/intra‐alveolar mucin. Using THG microscopy, these growth patterns and particular shape of the tumor cells were clearly revealed, in good agreement with the HE histology images. Besides tumor cells, the THG image also shows bright spots, which probably arise from lymphocyte cell nuclei. Lymphocytes have small and compact cell nuclei (visible as small dark purple dots in the HE image), and the presence of lymphocytes is an important prognostic and predictive factor in lung cancer, which defines the survival in resected patients (the more lymphocytes the better survival) [57, 58]. Therefore, it is of added value to visualize the lymphocytes using THG microscopy. A more complete image of the tumor growth pattern and the preexistent alveolar structures was obtained by generating a 3D projection (Figure 3C and Video S5) of a depth scan of 70 μm (Video S6).

An adenocarcinoma with also a lepidic growth pattern is shown in Figure 5E,F, with tumor cells lining the alveolar walls. The HE histology images (Figure 5E,F right) show that the alveoli are filled with cellular structures, which are (besides tumor cells) erythrocytes and alveolar macrophages. Figure 5F shows cellular structures with autofluorescent cytoplasm revealing the cell nuclei as dark holes, which are likely to be macrophages: cells with a diameter of 10 to 30 μm, and with eccentrically located, oval‐shaped nuclei [51]. In particular, these cells might be smokers' macrophages: cells with abundant cytoplasm and long wavelength autofluorescent signals (550‐650 nm) generated by accumulation of carbon particles or tar, as proposed by Jain et al. [25]. Furthermore, the preexistent alveolar honeycomb pattern of elastin and collagen are clearly observable in the THG/SHG/2PEF images, as expected in the adenocarcinoma with lepidic growth pattern whereas tumor cells grow along the preexistent alveolar walls.

In the last case shown in Figure 5G,H, intense THG signals are observed inside the alveoli, but clear contours of cell and cell nuclei are missing. Comparable nuclear morphology with very light chromatin pattern is also observed in the HE images of this tumor. This tumor case was a mixed lepidic and acinar adenocarcinoma, where the HE histology images show that the tumor cells lined the preexistent alveolar walls and induced an inflammatory reaction, which is a prognostic marker for lung cancer [59]. This is observable by the abundant number of inflammatory cells, mostly lymphocytes, which are visible in the HE histology image as small dark purple dots, as also observed in the mucinous adenocarcinoma case (Figure 5C,D). Similarly, in the THG/SHG/2PEF images bright spots are visible in the THG channel, which probably arise from the lymphocytes cell nuclei. Note that in contrast to the macrophages in Figure 5F, no 2PEF signal is detected from these lymphocytes, due to the different function of these two lineages in the lung: alveolar macrophages have a scavenger function and lymphocytes facilitate the immune reaction to the tumor. Furthermore, the high intensity THG signals make it more difficult to observe the contours of tumor cells and their cell nuclei. Besides the inflammatory reaction, also a fibrotic reaction is evident from the increased amount of collagen, originating from the desmoplastic stroma, a fibroblastic reaction to tumor invasion.

We conclude that the THG/SHG/2PEF images compare very well to the histology images and due to the correspondence of the visualized key histological features the images can be used by a pathologist to make a diagnosis of the tissue. The added value of THG to the imaging modalities is especially clear when imaging the epithelium and the tumor samples. These tissues generate strong THG signals that enables the visualization of cells. In principle, also 2PEF visualizes cells via endogenous fluorescence from NADH and FAD, see Jain et al. [25]. In our experiments, the THG signals were significantly stronger than the 2PEF signals from cells and, importantly, in several cases cells did not emit fluorescence while they did generate THG.

### 3.4 Translation into the clinic

To translate THG/SHG/2PEF microscopy into the clinic, it is important that the technique reveals pathological hallmarks with which nontumorous tissue can be distinguished from tumor tissue. For the lung, those histopathological hallmarks include general architecture (collagen and elastin organization) and cell morphology, which both can be revealed by THG/SHG/2PEF microscopy as discussed in the previous paragraphs. The THG/SHG/2PEF images showed even more information than the standard HE histology images, because elastin and collagen fibers are easily distinguished, for which in the histopathology an additional EVG staining is required. An EVG stain visualizes the amount and architecture of elastin and collagen deposition which are useful for the evaluation of (a) interstitial lung disease for evaluation and quantification of fibrosis and (b) tumor invasion and destruction of the elastic network of the normal lung parenchyma. When invasive, a tumor initiates a stromal fibroblastic proliferation, neoangiogenesis and collagen deposition which can be visualized using the SHG signal. Immediate visualization of the invasive tumor diameter would be of a significant value for patients' prognosis and treatment choice.

**FIGURE 6:**
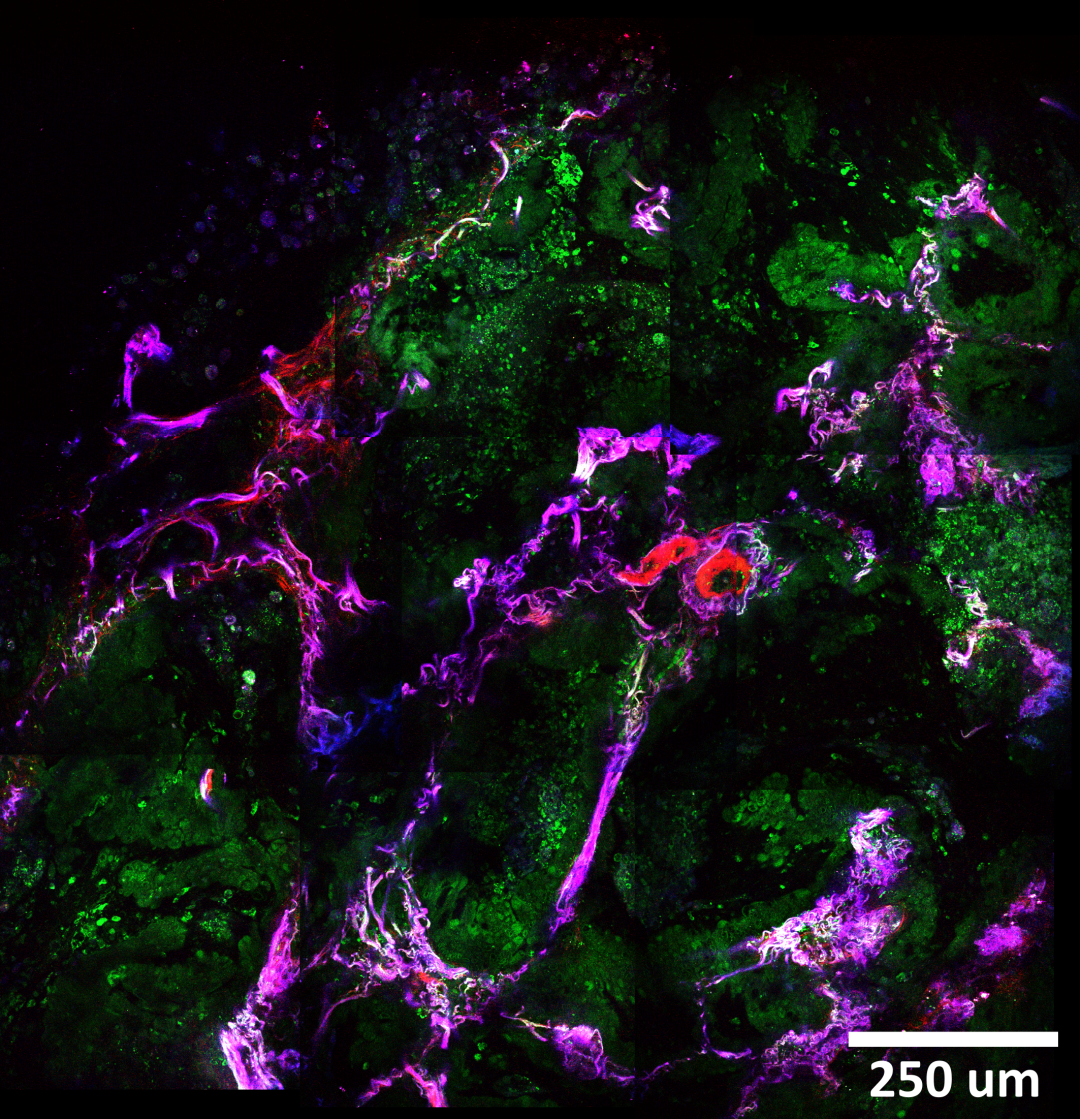
THG(green)/SHG(red)/2PEF(blue) mosaic image of mucinous adenocarcinoma tissue. An area of 1.2 × 1.2 mm^2^ is scanned by generating and stitching nine images with a field‐of‐view of 500 μm and an overlap of 10%, using an average power of ~5 mW. With an acquisition time of ~3.5 seconds per image, the total imaging time was ~32 seconds

Besides providing histopathological information, to be useful in the operation room it is also important that this information can be provided in a reasonable short time. The THG/SHG/2PEF images showed so far, were imaged with the “high‐quality” scanning mode and had an acquisition time of 3.5 seconds. However, the microscope also has a “inspection” mode, a scan program with an acquisition time of only 1 second. This “inspection” mode was used to scan through the tissue to have an overview of the sample and to seek for regions‐of‐interest. [Link], [Link], [Link] show examples of fast scanning through nontumorous peripheral lung tissue and two different types of tumor tissue. The quality of those fast‐scanned images is sufficiently to recognize the different alveolar structures and to select areas of interest for a closer examination. Another method to obtain an overview of the sample is by automatically scanning a larger area and stitching those images to generate a “mosaic” image. In combination with the “high‐quality” mode an area of 1.2 × 1.2 mm^2^ could be scanned in half a minute (Figure 6). Here, the acquisition seed is limited by the laser and sample scanning. Smarter designs employ a combination of laser scanning for *x*‐ and sample scanning for *y*‐direction, so‐called strip‐scanning [60, 61, 62]. Then, the acquisition speed limit lies at 1 mm^2^ per second, or 1 cm^2^ in less than 2 minutes, since our acquisition time is not limited by signal intensity, but by the repetition rate of the laser and acquisition electronics, which limits the data rate to 1 Mpixel per second. For a pixel size of 1 μm^2^, 10^6^ pixels would be required to scan an area of 1 mm^2^, or more for higher pixel densities. Improving the scanner design and device could therefore lead to an image acquisition speed for this label free technique close to the fastest nowadays achieved in fluorescence confocal microscopy, see for example the RS‐G4 device (mavig-research.com), which quotes an acquisition speed of 1 cm × 1 cm within 3 minutes with 6 Mpixels per second.

Furthermore, important to note is that the average power on the sample was only 5 mW. Subsequent histopathological analysis could reveal no changes or obvious damage to the tissue, and the tissue can therefore be used for further pathological analysis.

Upon translation into the clinic, the device may be used in the resection of centrally located lung tumors, and yield immediate feedback whether the excision border is free on malignancy, which will significantly reduce the operating time. In the endoscopy suite, analysis of tissue biopsies/smears of lung lesions/nodes can provide immediate feedback to the endoscopist thereby reducing investigation time and increasing diagnostic yield.

## 4 CONCLUSION

In this study we used a compact, portable microscope and recorded images within 1 to 3 seconds using a power of ~5 mW. Comparing the generated THG/SHG/2PEF images of tumorous and nontumorous tissues with the corresponding standard histology images shows that THG/SHG/2PEF microscopy is able to differentiate cellular structures, including tumor cells, macrophages and lymphocytes (THG), collagen (SHG) and elastin (2PEF). THG/SHG/2PEF images showed more information than the standard HE histology images, because elastin and collagen fibers are easily distinguished, for which in histopathology the additional EVG staining is required. This allows rapid identification of alveolar structures and histopathology hallmarks including cell morphology and general tissue architecture (collagen and elastin organization). THG/SHG/2PEF imaging is thus a promising tool for clinical intraoperative assessment of lung tumor tissue, which can lead to shorter transthoracic procedures, assessing surgical margins, acceleration of preoperative diagnostics and faster and safer CT‐guided and bronchoscopic diagnostics. To further investigate the added value of this imaging technique, the quality of diagnoses by pathologists based on these images needs to be assessed, similar to the study we recently conducted for brain tumors [45], and the clinical impact of the portable THG/SHG/2PEF microscope needs to be assessed in a clinical lung cancer setting. For the diagnosing and staging of lung cancer, its impact on bronchoscopic/transthoracic procedures needs to be evaluated. During lung cancer surgery, its intraoperative feedback should be explored taking patient management and related outcomes into account.

## CONFLICT OF INTEREST

Frank van Mourik and Marie Louise Groot declare to have, respectively, direct and indirect interest in Femto Diagnostics. Laura M. G. van Huizen received technical support from Femto Diagnostics. Femto Diagnostics was not involved in design of the study or analysis of the data.

## AUTHOR CONTRIBUTIONS

Laura M. G. van Huizen was involved in conceptualization, performing experiments, analyzing data, writing—original draft and project management. Marie Louise Groot was involved in conceptualization, writing—review and editing and project management. Frank van Mourik was involved in technical development and provided technical support. Danielle Seinstra was involved in providing samples, interpretation of data, writing—review and editing. Teodora Radonic was involved in conceptualization, interpretation of data, writing—review and editing. Chris Dickhoff was involved in providing samples and writing—review and editing. Johannes M. A. Daniels, Idris Bahce and Jouke T. Annema were involved in conceptualization, writing—review and editing.

## Supporting information


**Video S1** THG(green)/SHG(red)/2PEF(blue) depth scan of nontumorous peripheral lung tissue up to 70 μm deep.

**Video S2** THG(green)/SHG(red)/2PEF(blue) 3D projection of a depth scan of nontumorous peripheral lung tissue up to 70 μm deep, showing the 3D continuous elastin pattern in the alveolar wall.

**Video S3** THG(green)/SHG(red)/2PEF(blue) 3D projection of a depth scan of nontumorous bronchus wall lung tissue up to 80 μm deep, showing the 3D structure of cartilage tissue.

**Video S4** THG(green)/SHG(red)/2PEF(blue) depth scan of nontumorous bronchus wall lung tissue up to 100 μm deep, showing the different layers of the bronchus: respiratory epithelium (THG) and lamina propria, including collagen (SHG) and elastin (2PEF) fibers.

**Video S5** THG(green)/SHG(red)/2PEF(blue) 3D projection of a depth scan of tumorous lung tissue (mucinous adenocarcinoma with lepidic and micropapillary growth pattern) up to 70 μm deep.

**Video S6** THG(green)/SHG(red)/2PEF(blue) depth scan of tumorous lung tissue (mucinous adenocarcinoma with lepidic and micropapillary growth pattern) up to 70 μm deep.

**Video S7** Real‐time imaging with THG(green)/SHG(red)/2PEF(blue) microscopy through nontumorous peripheral lung tissue. Acquisition time per image is ~1 second.

**Video S8** Real‐time imaging with THG(green)/SHG(red)/2PEF(blue) microscopy through tumorous lung tissue (typical carcinoid with solid growth pattern), showing the transition area from collapsed alveolar tissue to the tumor area. Acquisition time per image is ~1 second.

**Video S9** Real‐time imaging with THG(green)/SHG(red)/2PEF(blue) microscopy through tumorous lung tissue (mucinous adenocarcinoma with lepidic and micropapillary growth pattern). Acquisition time per image is ~1 second.

## Data Availability

The data that support the findings of this study are available on request from the corresponding author.
